# Treatment persistence to tolvaptan in patients with autosomal dominant polycystic kidney disease: a secondary use of data analysis of patients in the IMADJIN® dataset

**DOI:** 10.1186/s12882-021-02607-4

**Published:** 2021-12-02

**Authors:** Mark Thomas, Pedro Henrique Franca Gois, Belinda E. Butcher, Michelle H. T. Ta, Greg W. Van Wyk

**Affiliations:** 1 Department of Nephrology, Royal Perth Hospital, Perth, WA Australia; 2 Department of Nephrology, Fraser Coast Hospital and Health Service, Hervey Bay, QLD Australia; 3 University of Queensland, School of Medicine, Brisbane, QLD Australia; 4 WriteSource Medical Pty Ltd, Lane Cove, NSW Australia; 5 School of Medical Sciences, University of New South Wales, UNSW, Sydney, NSW Australia; 6 Otsuka Australia Pharmaceutical Pty Ltd, Chatswood, NSW Australia; 7 AIH Consulting Pty Ltd, Westleigh, NSW Australia

**Keywords:** Autosomal dominant polycystic kidney disease, Medication persistence, Tolvaptan, Australia, Real world evidence

## Abstract

**Background:**

Tolvaptan is the only available disease-modifying treatment for autosomal dominant polycystic kidney disease (ADPKD). Prior to October 2020 access to tolvaptan in Australia was restricted by a controlled monitoring and distribution program called IMADJIN®. Focusing on hepatic safety, the IMADJIN® program collected real-world data on patients with ADPKD. A retrospective, secondary data analysis of the IMADJIN® dataset was undertaken to determine the time to all-cause discontinuation of tolvaptan in Australia.

**Methods:**

Demographic and treatment data from 17 September 2018 to 30 September 2020 were extracted from the IMADJIN® dataset. Treatment persistence was analyzed using Kaplan-Meier methods, and Cox’s proportional hazard models were used to analyze differences in treatment persistence by age, sex and location.

**Results:**

Four hundred seventy-nine patients with ADPKD were included in the analysis. After a median follow-up of 12.0 months (95% confidence interval [CI] 2.6, 23.4), the Kaplan-Meier estimation of 12-month persistence was 76.7% (95% CI 72.2, 80.5%). 114 (23.8%) patients discontinued treatment; sex, state, and remoteness did not significantly affect treatment persistence. Patients in the youngest tertile were more likely to discontinue compared to older ages (*p* = 0.049). Reasons for discontinuation included: aquaretic tolerability (4.2%), hepatic adverse events (abnormal liver function tests) (2.1%), disease progression (1.5%), and acute kidney injury (0.2%). Patients with a lack of aquaretic tolerance had shorter time to discontinuation. Hepatic toxicity events were initially observed 3 months after tolvaptan initiation and were less prevalent over time.

**Conclusions:**

Persistence to tolvaptan in the real-world IMADJIN® dataset was 76%. Discontinuation due to hepatic events was low. Prescribers should take extra care when initiating treatment in younger patients as they are more likely to discontinue tolvaptan compared to older individuals. Nevertheless, the precise reason for this observation remains to be elucidated.

**Supplementary Information:**

The online version contains supplementary material available at 10.1186/s12882-021-02607-4.

## Background

Autosomal dominant polycystic kidney disease (ADPKD) is the most prevalent inherited progressive kidney disease [1], affecting between 3.96 and 10 in 10,000 Australians [2, 3], and is the most common genetic cause of renal failure in adults. ADPKD is characterized by the development of cysts within the kidney impacting on the kidney architecture leading to gradual loss of function which often progresses to end-stage renal disease [1]. Extrarenal manifestations such as polycystic liver disease, arachnoid cysts and intracranial saccular aneurysms also occur [4].

Treatment has been limited to reducing the morbidity and mortality associated with the complications of ADPKD including hypertension, pain, cyst hemorrhage and infection, and end stage renal disease. More recently, tolvaptan, the first disease-modifying treatment for ADPKD, has been registered in a number of countries including Australia. Tolvaptan is a selective vasopressin V2-receptor antagonist that has been shown in two large phase 3 randomized controlled trials in early and more advanced ADPKD (TEMPO 3:4 and REPRISE) to slow the increase in total kidney volume and lower the rate of decline in estimated glomerular filtration rate (eGFR) compared to placebo [5, 6].

The Phase 3 TEMPO 3:4 trial [6] enrolled 1445 patients with ADPKD patients with stage 1 to 3 chronic kidney disease, total kidney volume at least 750 mL and estimated creatinine clearance at least 60 mL/min and involved 36 months of tolvaptan dosing. The benefit in kidney function seen in this study was maintained during the two-year open label extension study TEMPO 4:4 [7]. Common adverse events with higher incidence in patients receiving tolvaptan than placebo included polyuria, nocturia, thirst, polydipsia, dry mouth, diarrhea, and fatigue. In the TEMPO 3:4 trial, 23% of patients in the tolvaptan arm discontinued, mainly due to aquaresis or hepatic anomalies, compared to 14% in the placebo arm. There were three cases of potentially serious drug-induced liver injury meeting Hy’s law in the TEMPO trials.

The Phase 3 REPRISE trial [5] in 1495 patients with stage 2 to early stage 4 chronic kidney disease, included more frequent monitoring for toxic effects in the liver. In this study, there were no cases of potentially serious drug-induced liver injury meeting Hy’s Law. Elevation of alanine aminotransferase above three times the upper limit of the normal range occurred in 38 patients (5.6%) in the tolvaptan arm and 8 (1.2%) of the placebo arm. These elevations were reversible on ceasing tolvaptan therapy.

The tolvaptan long-term, open label, extension study in 1803 patients who had previously completed any tolvaptan trial included monthly liver function testing during the first 18 months of tolvaptan therapy and every 3 months thereafter [8]. The median tolvaptan exposure during the extension study was 651 days (interquartile range 538 to 924), and the cumulative exposure from this and previous trials of tolvaptan was up to 11 years [8]. The study did not identify any new safety issues and demonstrated that regular and ongoing monitoring of hepatic function in patients treated with tolvaptan enabled early and effective intervention [8].

In 2017, tolvaptan was approved in Australia for the indication of slowing the progression of cyst development and renal insufficiency of ADPKD in adults with stage 1 to 3 chronic kidney disease (CKD) at initiation of treatment and evidence of rapidly pressing disease [9]. After becoming available for prescription in September 2018, access to tolvaptan in Australia was restricted until 30 September 2020, as part of a controlled monitoring and distribution program called IMADJIN®. The primary objective of this program was to monitor hepatic safety. Patients receiving tolvaptan are required to undergo liver function testing (LFT) including alanine aminotransferase (ALT), aspartate aminotransferase (AST) and total bilirubin (BT) prior to commencing treatment, monthly for the first 18-months, and three-monthly thereafter [9]. We note that while treating clinicians were able to review detailed pathology reports for their patients’ LFTs, the IMADJIN® dataset only collected data on the outcome of the results (‘normal’ or ‘abnormal’) and tolvaptan treatment decision (‘continue treatment’ or discontinue treatment’) at each timepoint.

The IMADJIN® program also provided the opportunity to study tolvaptan in a real-world setting. Persistence to tolvaptan therapy is an area of interest considering the discontinuation rate observed in the TEMPO 3:4 trial due to aquaretic side effects. Studies have shown a variation in treatment discontinuation rates in patients treated with loop diuretics for heart failure [10], and patients treated with diuretics are more likely to have changes made to their treatment regimen. There are currently limited real-world studies of tolvaptan, although the Canadian C-MAJOR study of tolvaptan has also investigated treatment persistence and hepatic safety [11].

The primary objective of this study was to determine treatment persistence in patients prescribed tolvaptan through the IMADJIN® program. Secondary objectives included to describe the IMADJIN® program patient demographics, to determine the rate of tolvaptan treatment discontinuation, to identify any differences in treatment persistence based on demographic characteristics, to identify the proportion of patients with treatment interruptions and to determine changes in liver function test results. Further we sought to identify reasons for discontinuation, any association between treatment dose and discontinuation, and any association between meteorological season and discontinuation.

## Methods

We conducted a retrospective, secondary analysis of data held in the IMADJIN® dataset. Ethics approval and a waiver of informed consent to use de-identified data from the IMADJIN® dataset was granted by the Royal Perth Hospital Human Research Ethics Committee, Perth, Western Australia (approval number: RGS0000003858).

### Patient population

De-identified data from individuals initiated on tolvaptan from 17 September 2018 to 30 September 2020 were extracted from the IMADJIN® program dataset. All eligible patients within the IMADJIN® dataset were included in the analysis. That is, patients who commenced tolvaptan in a clinical trial setting were excluded from the analysis as their treatment was initiated prior to the establishment of the IMADJIN® program and treatment was not under observational conditions. Patients who were enrolled in the IMADJIN® program but who never received tolvaptan treatment were also excluded. Additional information on the IMADJIN® program and patient enrolment criteria is provided as a supplementary file (Supplementary File 1).

### Data extraction

Extracted data included: age, sex, state of residence, Australian Bureau of Statistics (ABS) remoteness category [12], tolvaptan initiation and permanent discontinuation date, and reason for treatment discontinuation. Dose and safety data, including LFT results, were extracted for each follow-up interval (monthly for the first 18-months and quarterly thereafter).

### Statistical analysis

All analyses were performed on the full available dataset and no imputation of missing data occurred. Treatment persistence is defined as the time in consecutive days from the date of enrolment in the IMADJIN® program, until the date of discontinuation in the program. Patients who continued in the program were censored on 30 September 2020. For the purposes of analysis, age was divided into tertiles containing approximately equal numbers of patients in each tertile (18–43, 44–54 and 55–77 years of age).

At the time of data collection, the IMADJIN® program coordinator was able to select from pre-specified categories for reasons for discontinuation which included ‘aquaretic tolerability’ and ‘disease progression’. Additional information could also be captured in free text fields, but this was not universal. Where a reason did not fit one of the pre-specified categories, the IMADJIN® program coordinator selected ‘other’. Examples of aquaretic tolerability may have included polyuria, nocturia or pollakiuria.

Treatment persistence was estimated using Kaplan-Meier methods and differences in treatment persistence based on age, sex, state and remoteness were analyzed using Cox’s proportional hazard models. Logistic regression was used to investigate if there was any association between dose escalation and treatment discontinuation. All other analyses were descriptive. Analyses were conducted in Stata MP v16.2 for Mac (StataCorp, Texas Station, US). Values of *p* < 0.05 were considered statistically significant.

## Results

Of the 542 enrolments in the IMADJIN® dataset, a total of 63 patients were excluded from this analysis (*n* = 46 clinical trial participants, *n* = 17 who did not commence treatment). Therefore data from 479 patients were extracted from the IMADJIN® dataset; demographic data for the 479 patients are presented in Table 1.

**Table 1:** Demographic characteristics

	Factor	Patients, n(%) *N* = 479	Australian Population by State^**a**^ (%)
Age (years, mean (SD))		49.6 (11.6)	
Age category (years)	< 25	7 (1.5%)	
	25–34	35 (7.3%)	
	35–44	128 (26.7%)	
	45–54	145 (30.3%)	
	55–64	100 (20.9%)	
	> = 65	64 (13.4%)	
Age tertile	Lowest age tertile (18–43 years)	150 (31.3%)	
	Middle age tertile (44–54 years)	165 (34.4%)	
	Highest age tertile (55–77 years)	164 (34.2%)	
Sex	Male	265 (55.3%)	
	Female	212 (44.3%)	
	Missing	2 (0.4%)	
State	Australian Capital Territory (ACT)	17 (3.5%)	1.7%
	New South Wales (NSW)	119 (24.8%)	31.8%
	Northern Territory (NT)	6 (1.3%)	0.9%
	Queensland (QLD)	125 (26.1%)	20.1%
	South Australia (SA)	9 (1.9%)	6.9%
	Tasmania (TAS)	16 (3.3%)	2.1%
	Victoria (VIC)	148 (30.9%)	26.1%
	Western Australia (WA)	39 (8.1%)	10.4%
Remoteness Category	Major Cities of Australia	332 (69.3%)	
	Inner Regional Australia	108 (22.5%)	
	Outer Regional Australia	38 (7.9%)	
	Remote Australia	1 (0.2%)	

*ABS* Australian Bureau of Statistics

^a^ data at 30 June 2020 [13]

### Tolvaptan dosing

Approximately half of patients never escalated beyond a daily dose of 60 mg (as a split dose of one 45 mg plus one 15 mg) (54.3%). Approximately 1 in 5 were up-titrated to 90 mg (as a split dose of one 60 mg plus one 30 mg) (21.8%) and remained on that dose, whereas a further quarter up-titrated to 120 mg (as a split dose of one 90 mg plus one 30 mg) (24.0%). On average, doses were up-titrated to 90 mg after 5.4 ± 3.3 months from commencement of treatment, and to 120 mg after 6.8 ± 3.2 months from commencement of treatment.

### Treatment persistence

**Fig. 1:**
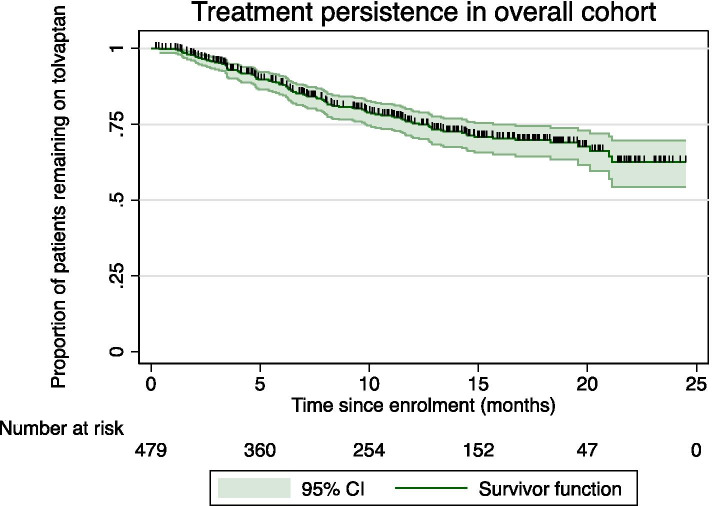
Treatment persistence in overall cohort

**Fig. 2:**
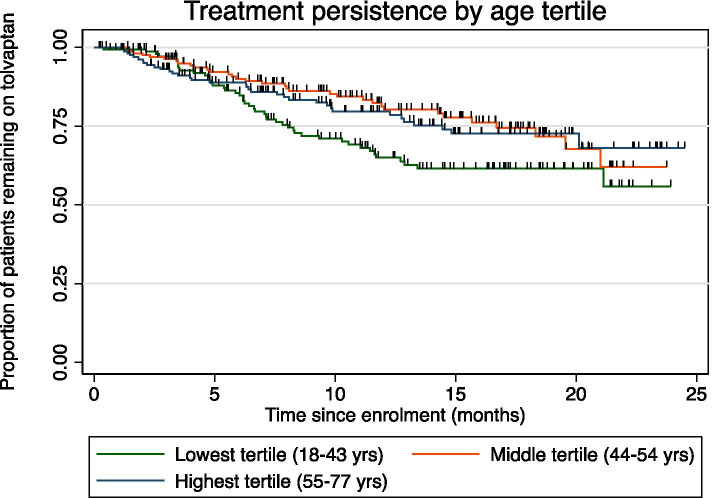
Treatment persistence by age tertile. Ticks represent censoring

After a median follow-up of 12.0 months (95% confidence interval [CI] 2.6, 23.4), 12-month treatment persistence was 76.7% (95% CI 72.2, 80.5%). Overall, 114 patients (23.8%) discontinued treatment (Fig. 1). Treatment persistence was not significantly affected by sex, state or remoteness. Age was found to have a significant impact on treatment persistence, with patients in the youngest tertile being more likely to discontinue treatment early compared to patients in the middle or oldest tertiles (*p* = 0.049). There was no difference in treatment persistence between the middle and oldest tertiles (Fig. 2).

### Treatment discontinuation

The overall discontinuation rate was 231.7 per 1000 patient years. Reasons for discontinuation included healthcare professional/ patient decision, not otherwise specified (5.9%), aquaretic tolerability (4.2%), hepatic adverse events (abnormal LFTs) (2.1%), and disease progression (1.5%) (Table 2). When adjusted for maximum dose and age, compared to other reasons, those with a lack of aquaretic tolerability had a significantly shorter time to discontinuation (median 4.8 months compared to 6.7 months, HR 1.92, 95% CI 1.02 to 3.61, *p* = 0.04). Escalation of tolvaptan treatment to either 90 mg or 120 mg per day did not appear to be associated with early treatment discontinuation. The effects of season on discontinuation is reported in the supplementary text.

**Table 2:** Reason for tolvaptan discontinuation (*n* = 114)

Reason for discontinuation	n (%^a^)
Abnormal LFTs	10 (2.1)
Acute renal impairment	1 (0.2)
Adverse event, NOS	2 (0.4)
Aquaretic tolerability	20 (4.2)
Disease progression	7 (1.5)
HCP/ patient decision, NOS	28 (5.9)
Non-hepatic adverse event, NOS	12 (2.5)
Treatment break – failed to restart treatment	11 (2.3)
Patient lost to follow up	10 (2.1)
Other	13 (2.6)

*HCP* healthcare professional; *LFT* Liver function test; *NOS* not otherwise specified

^a^Percentage of the total population

### Safety

**Fig. 3:**
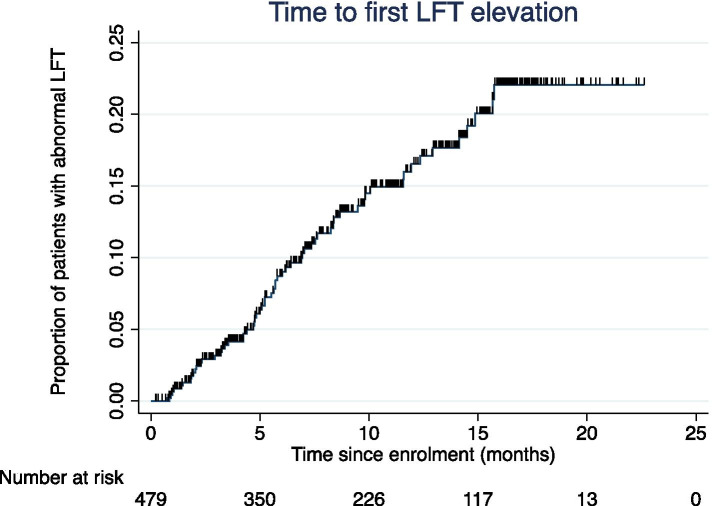
Time to first liver function test (LFT) elevation. Ticks represent censoring

**Fig. 4:**
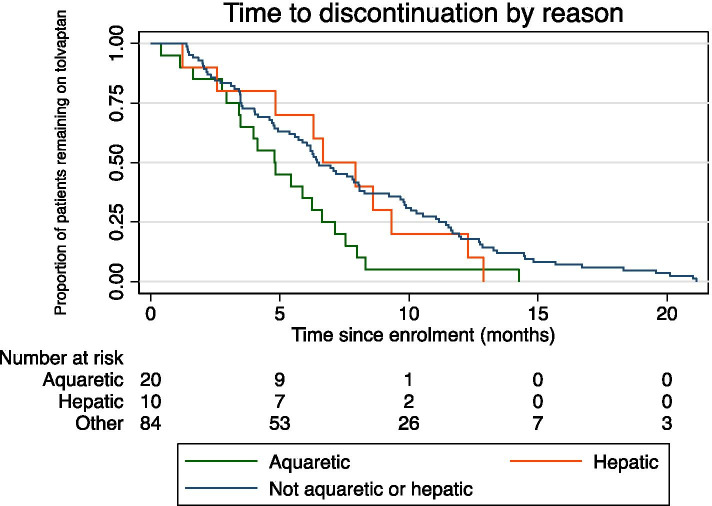
Time to discontinuation for aquaretic events, hepatic events, and other discontinuations

Of patients with at least one recorded LFT result (*n* = 437), 14% reported an abnormality at some point during the period of analysis (Fig. 3). Abnormal LFTs resulted in discontinuation of tolvaptan in 2.1% of patients. High rates of missing data were noted for clinicians’ determination of whether LFTs were normal or abnormal. The rate of hepatic and other reported side effects were not tolvaptan dose related, nor were they related to the length of time a patient was treated with tolvaptan. Time to discontinuation for aquaretic events, hepatic events, and other discontinuations are presented in Fig. 4. Compared to other reasons, the time to discontinuation was significantly shorter in those experiencing aquaretic tolerability effects (adjusted HR 1.92 (95% 1.02 to 3.6); adjusted for dose and age). One event of acute renal impairment was recorded; however, it is unclear from the available data if this was due to disease progression or another cause.

## Discussion

This study presents the first data on tolvaptan use in Australia outside of a clinical trial setting and provides key insights into the real-world use of this treatment in patients with ADPKD. The real-world tolerability of tolvaptan was similar to that seen in the pivotal trials [6, 7] and is similar to other medications used in the treatment of chronic kidney disease, such as anti-hypertensives [14–16]. Real world data on treatment persistence and tolerability is important given the differences between the tolvaptan clinical trial entry criteria and those eligible for reimbursed treatment in Australia under Pharmaceutical Benefits Scheme (PBS) criteria. Currently, to qualify for PBS reimbursement, patients must have an eGFR between 30 and 89 mL/min/1.73m^2^ and evidence of rapidly progressing disease at the time of initiation of tolvaptan (defined as either decline in eGFR ≥5 mL/min/1.73m^2^ within 1 year, or an average decline in eGFR ≥2.5 mL/min/1.73m^2^ per year over a five-year period) [17]. We note therefore that the population included in this analysis may be different to that included in the tolvaptan clinical trials.

The proportion of patients who discontinued in our study (23.8%), were similar to that reported in TEMPO 3:4 study (23%) [6], but higher than the discontinuation rates in the REPRISE study (5% during the run-in period plus 9.5% during the double-blind period) [5] and the Canadian real-world C-MAJOR study (16.8%) [11]. Similar to other studies, tolvaptan discontinuations occurred steadily over time, although the rate of discontinuation does appear to slow after 12-months. While the odds (risk) of discontinuing during summer compared to other seasons was higher, no difference in the reason for discontinuation by season were noted, despite a theoretical possibility of increased discontinuation due to aquaretic tolerability issues (such as thirst) during the warmer months. Due to the retrospective nature of this study, we were unable to further categorise the true reasons for treatment discontinuation or undertake a root cause analysis relating to reasons for discontinuation. We note that the categories ‘HCP/patient decision, not otherwise specified’ (5.9%), ‘treatment break – failed to restart treatment’ (2.3%) and ‘lost to follow-up’ (2.1%) presented in Table 2 may reflect latent tolerability issues in some patients.

Younger patients were more likely to discontinue treatment compared to older patients, the reason for which is unclear. Younger patients may have better preserved renal function, and higher baseline urine osmolality, and therefore a stronger aquaretic response to tolvaptan [18] as shown in prior studies. A second hypothesis is that due to their younger age, and as they are healthier, their persistence to medication is a lesser priority given the reality of renal replacement therapy is ‘in the distant future’. Other reasons might include less awareness of disease progression, or greater physical activity leading to more aquaretic tolerability issues. These hypotheses should be explored in future clinical studies.

The rates of hepatic injury (as measured by an abnormal LFT) were low, as were discontinuations due to hepatic injury, with twice as many patients discontinuing treatment due to aquaretic effects compared to hepatic effects. The pattern of hepatic toxicity in this study was similar to that seen in other studies [8, 11] where the occurrence of events attenuates over time [19]. After an early elevation in the rate of abnormal LFTs, the rate decreased over time; we note that the temporal pattern of the trend in the IMADJIN® study is different to that seen in the pivotal tolvaptan trials. As suggested, aquaretic tolerability may differ with age, with older patients less likely to discontinue treatment due to these effects. Our study is limited by missing data entry for LFTs which was approximately 25% throughout the study.

Those treated on maximum dose of tolvaptan (120 mg) were more likely to continue treatment when compared to those on lower doses. Despite the prescriber’s ability to titrate tolvaptan to 120 mg per day [9], over half of the patients in this study remained on tolvaptan 60 mg, which is similar to that seen in other real world studies of tolvaptan [11]. Reasons for remaining on the lower dose of tolvaptan are unknown, but may be due to prescriber inertia or aquaretic intolerance of higher doses by the patient. The TEMPO 3:4 and REPRISE studies reported the average dose of tolvaptan to be between 90 mg to 120 mg [5, 6], and a long-term extension study found the average daily dose of tolvaptan to be 96 mg [8]. A phase 2 study suggested that patients treated with higher doses of tolvaptan had greater treatment effects, as measured by total kidney volume, compared to those on lower doses [9]. The average dose changes over time are noteworthy as they suggest a more cautious approach to dose escalation than was followed in the tolvaptan registration studies [5, 6]. However, significant inter-individual variability in dose adjustments was observed perhaps indicating wide variation in Australian clinicians’ approaches to dosing tolvaptan.

We were unable to confirm the relationship between tolvaptan dose and kidney growth in our study due to a lack of data on total kidney volume (TKV) or renal function. As described earlier, since TKV does not form part of the criteria for reimbursement in Australia, data on TKV is not readily available and therefore not captured in this study.

Of interest are differences in tolvaptan prescribing by Australian State and Territory (Table 1). The greatest proportion of IMADJIN® program participants were enrolled in Victoria (31%) while in both absolute terms and on a per capita basis a low proportion of participants were enrolled from South Australia (1.9% of IMADJIN patients vs. 6.9% of the Australian population) (Table 1). The reason behind the wide variation in state-to-state tolvaptan prescribing rates remains uncertain.

### Limitations

Limited demographic and treatment data were collected on patients treated with tolvaptan via the IMADJIN® program. Further, the secondary analysis of real-world data is limited by incomplete or missing data. Hepatic safety was monitored routinely and was the primary focus of the IMADJIN® program, therefore other reasons for discontinuation may not have been collected as comprehensively. Due to a lack of renal function data we were unable to observe the effects of tolvaptan on renal function decline, or quantify the magnitude of abnormal LFTs as the data only reported ‘normal’ or ‘abnormal’ for this data point. Finally, no data on TKV were collected in the IMADJIN® dataset and therefore the efficacy of tolvaptan in slowing kidney growth could not be determined in this study. Finally, no data on TKV were collected in the IMADJIN® dataset. We note that TKV is not mentioned as a requirement for identifying patients at risk of rapid progression, by either the Australian regulator (Therapeutic Goods Administration) or the payer (PBS), and to the best of our knowledge Australian nephrologists do not routinely use TKV to risk stratify patients.

## Conclusions

Australian real-world tolvaptan data show similar treatment tolerability to that seen in the pivotal studies. Low hepatic adverse events were noted in this patient population. The variation in tolvaptan discontinuation by age and by season suggest that careful counselling and monitoring of patients should be considered, especially during the summer months and for those patients who are younger in age.

## Supplementary Information

**Additional file 1.**

## Data Availability

The datasets generated and/or analysed during the current study are not publicly available due to limitations imposed by the Health Research Ethics Committee. For data requests please contact the corresponding author.

## Abbreviations

ABS: Australian Bureau of Statistics
ADPKD: Autosomal dominant polycystic kidney disease
ALT: Alanine aminotransferase
AST: Aspartate aminotransferase
BT: Bilirubin total
CKD: Chronic kidney disease
eGFR: Estimated Glomerular Filtration Rate
LFT: Liver function tests
TGA: Therapeutic Goods Administration
TKV: Total kidney volume
PBS: Pharmaceutical Benefits Scheme

## References

[1] Bergmann C, Guay-Woodford LM, Harris PC, Horie S, Peters DJ, Torres VE (2018). Polycystic kidney disease. Nature Reviews Disease Primers.

[2] PKD Australia. What is ADPKD? Access date: 04 February 2021. Available at: https://pkdaustralia.org/adpkd/

[3] Willey CJ, Blais JD, Hall AK, Krasa HB, Makin AJ, Czerwiec FS (2017). Prevalence of autosomal dominant polycystic kidney disease in the European Union. Nephrol Dial Transplant.

[4] Torres VE, Harris PC, Pirson Y (2007). Autosomal dominant polycystic kidney disease. Lancet.

[5] Torres VE, Chapman AB, Devuyst O, Gansevoort RT, Perrone RD, Koch G, Ouyang J, McQuade RD, Blais JD, Czerwiec FS (2017). Tolvaptan in later-stage autosomal dominant polycystic kidney disease. N Engl J Med.

[6] Torres VE, Chapman AB, Devuyst O, Gansevoort RT, Grantham JJ, Higashihara E, Perrone RD, Krasa HB, Ouyang J, Czerwiec FS (2012). Tolvaptan in patients with autosomal dominant polycystic kidney disease. N Engl J Med.

[7] Torres VE, Chapman AB, Devuyst O, Gansevoort RT, Perrone RD, Dandurand A, et al. Multicenter, open-label, extension trial to evaluate the long-term efficacy and safety of early versus delayed treatment with tolvaptan in autosomal dominant polycystic kidney disease: the TEMPO 4:4 trial. Nephrol Dial Transplant. 2017;33(3):477-489. 10.1093/ndt/gfx043 PMC6019005 28379536

[8] Torres VE, Chapman AB, Devuyst O, Gansevoort RT, Perrone RD, Lee J, Hoke ME, Estilo A, Sergeyeva O: Multicenter Study of Long-Term Safety of Tolvaptan in Later-Stage Autosomal Dominant Polycystic Kidney Disease. Clin J Am Soc Nephrol 2021. 10.2215/CJN.10250620 PMC7792652 33376102

[9] Otsuka Pharmaceutical Australia Pty Ltd. Australian Product Information JINARC® (tolvaptan) tablets Access date: 06 January 2021. Available at: www.ebs.tga.gov.au/

[10] MacFadyen RJ, Gorski JC, Brater DC, Struthers AD (2004). Furosemide responsiveness, non-adherence and resistance during the chronic treatment of heart failure: a longitudinal study. Br J Clin Pharmacol.

[11] McFarlane P, Parfrey P, Bichet DG, Bergeron L, Laplante A. Abstract PO1537 Canadian real-world assessment of Tolvaptan in ADPKD: C-Major study and safety monitoring and distribution program. American Society of Nephrology Kidney Week. Digital Meeting 2020.

[12] Australian Bureau of Statistics. The Australian Statistical Geography Standard (ASGS) remoteness structure Access date: 18 May 2021. Available at: https://www.abs.gov.au/websitedbs/D3310114.nsf/home/remoteness+structure

[13] Australian Bureau of Statistics. National, state and territory population Access date: 04 February 2021. Available at: https://www.abs.gov.au/statistics/people/population/national-state-and-territory-population/jun-2020

[14] Truong VT, Moisan J, Kröger E, Langlois S, Grégoire J-P (2016). Persistence and compliance with newly initiated antihypertensive drug treatment in patients with chronic kidney disease. Patient preference and adherence.

[15] Gor D, Lee TA, Schumock GT, Walton SM, Gerber BS, Nutescu EA, Touchette DR (2020). Adherence and persistence with DPP-4 inhibitors versus pioglitazone in type 2 diabetes patients with chronic kidney disease: a retrospective claims database analysis. Journal of Managed Care & Specialty Pharmacy.

[16] Godino C, Melillo F, Rubino F, Arrigoni L, Cappelletti A, Mazzone P, Mattiello P, Della Bella P, Colombo A, Salerno A (2019). Real-world 2-year outcome of atrial fibrillation treatment with dabigatran, apixaban, and rivaroxaban in patients with and without chronic kidney disease. Intern Emerg Med.

[17] Deapartment of Health Australian Government. Tolvaptan PBS Listing Access date: 01 April 2021. Available at: https://www.pbs.gov.au/medicine/item/11600M-11602P

[18] Devuyst O, Chapman AB, Shoaf SE, Czerwiec FS, Blais JD (2017). Tolerability of Aquaretic-related symptoms following Tolvaptan for autosomal dominant polycystic kidney disease: results from TEMPO 3:4. Kidney Int Rep.

[19] Watkins PB, Lewis JH, Kaplowitz N, Alpers DH, Blais JD, Smotzer DM, Krasa H, Ouyang J, Torres VE, Czerwiec FS (2015). Clinical pattern of tolvaptan-associated liver injury in subjects with autosomal dominant polycystic kidney disease: analysis of clinical trials database. Drug Saf.

