# Supplementary material for: Treatment persistence to tolvaptan in patients with autosomal dominant polycystic kidney disease: a secondary use of data analysis of patients in the IMADJIN® dataset
Source: BMC Nephrol. 2021 Dec 2;22:400. doi: 10.1186/s12882-021-02607-4 (PMC8638092; doi:10.1186/s12882-021-02607-4)
Supplement: Supplementary file 1 — Additional file 1. [file 12882_2021_2607_MOESM1_ESM.docx]

**SUPPLEMENTARY TEXT**

**The IMADJIN^®^ Program: Patient Enrolment Criteria and Data Collection**

IMADJIN^®^ was established as a program to enable controlled supply of tolvaptan to every patient in Australia who was issued with a valid prescription for tolvaptan. While patients could self-fund their treatment and be supplied through the program, all patients in the program who went on to start tolvaptan treatment received treatment via the Pharmaceutical Benefits Scheme (PBS), Australia’s national medicines reimbursement scheme (https://www.pbs.gov.au/).

In Australia to be eligible for subsidised treatment with tolvaptan the patient’s condition must fulfil the following criteria:

- They must be diagnosed with autosomal dominant polycystic kidney disease (ADPKD)

**Treatment criteria:**

- Must be treated by a nephrologist.

**Clinical criteria:**

- The patient must have an estimated glomerular filtration rate (eGFR) between 30 mL/min 1.73 m^2^ and 89 mL/min 1.73 m^2^ at the initiation of treatment.

**AND**

- The patient must have or have had rapidly progressing disease at the time of initiation.

Rapidly progressing disease is defined as either of the following:

- A decline in eGFR of greater than or equal to 5 mL/min/1.73 m^2^ within one year;

**OR**

An average decline in eGFR of greater than or equal to 2.5 mL/min/1.73 m^2^ per year over a five-year period.

No patients were excluded from enrolment in the IMADJIN^®^ program. Enrolment was entirely at the discretion of the treating clinician.

To receive each month’s supply of tolvaptan, the patient’s treating clinician was required to confirm that the patient had undergone liver function (LFT) testing that month, that they had reviewed the results and they had decided to continue treatment.

If abnormalities were observed by the clinician this was captured in the IMADJIN^®^ dataset– only “normal” or “abnormal” were reported.

The management of adverse effects such as LFT abnormalities was at the treating clinician’s discretion.

If in any given month a decision was taken to cease treatment, whether by the clinician or the patient themselves, the IMADJIN^®^ program coordinator would record the reason for discontinuation, as defined by the clinician/patient. Where this reason correlated with one of the pre-specified categories such as “Aquaretic tolerability” or “Disease progressed”, it was captured as such. Due to the set-up of the system, only one reason for discontinuation could be selected per discontinuation (i.e. multiple reasons for discontinuation could not be selected for the same patient). Additional detail could also be captured in free text fields, but this was not systematically done. Where in the opinion of the IMADJIN^®^ program coordinator the reason did not fit one of these categories “Other” was selected and limited free text notes were captured.^[[1]](#footnote-2)^

Dose adjustment, laboratory tests other than LFTs, imaging, and other clinical management was at the treating clinician’s discretion. Of these, only dose adjustment was captured in the IMADJIN^®^ dataset. Reasons for dose adjustment were not explicitly captured.

**Seasonal Variations in Discontinuations**

Meteorological season was calculated at the date of discontinuation or the date of the last dose, with the full months of December, January and February considered as summer, March, April, May as autumn etc. Logistic regression was used to investigate if there was any association between meteorological seasons and treatment discontinuation.

There were significant differences in the odds of discontinuation by season (n=465; likelihood ratio Chi squared test with 4 degrees of freedom = 254.11, p<0.0001; Supplementary Table 1)

Supplementary Table 1: Logistic regression of seasonal differences in discontinuation

| Parameter | Odds Ratio | 95% CI | p-value |
| --- | --- | --- | --- |
| Season |  |  |  |
| Autumn | 0.328 | 0.050, 2.114 | 0.240 |
| Winter | 0.058 | 0.010, 0.354 | 0.002 |
| Spring | 0.004 | 0.007, 0.026 | <0.001 |
| Length on treatment | 0.743 | 0.696,0.793 | <0.001 |
| Constant | 376.8 | 51.02, 2783.6 | <0.001 |

Note: Season reference category is Summer. Constant estimates the baseline odds

Compared to summer, patients were significantly less likely to discontinue treatment during winter (OR [odds ratio] 0.06, 95%CI 0.01 to 0.35, p<0.002) or spring (OR 0.004, 95%CI 0.0007 to 0.03, p<0.001). There was a trend towards patients being less likely to discontinue in autumn compared to summer (OR 0.33, 95% CI 0.05 to 2.11, p=0.24).

Differences in the incidence of drug discontinuations may be multifactorial, and the results should be interpreted with caution given dates were used to classify seasons, without consideration of different seasonal effects. Further, the model fit was assessed and found to be poor suggesting there were missing covariates within the model which may influence discontinuation.

1. During preparation of the data for analysis, some reasons for discontinuation that were categorised as “other” were recoded based on the free text in the patient’s record e.g. “Other” – Nocturia” or “Other – cannot tolerate water” were recoded as “Aquaretic Tolerability”. [↑](#footnote-ref-2)
